# The impact of delayed tracheostomy on critically ill patients receiving mechanical ventilation: a retrospective cohort study in a chinese tertiary hospital

**DOI:** 10.1186/s12871-024-02411-1

**Published:** 2024-01-23

**Authors:** Jie Zhao, Wei Zheng, Nan-xian Xuan, Qi-chao Zhou, Wei-bing Wu, Wei Cui, Bao-ping Tian

**Affiliations:** 1 Department of Critical Care Medicine, The Second Affiliated Hospital, Zhejiang University School of Medicine, 88 Jiefang Rd, Hangzhou, 310009 China; 2 Department of Critical Care Medicine, The First Affiliated Hospital, Ningbo University, Ningbo, Zhejiang China; 3 Department of Critical Care Medicine, Zhejiang Daishan First People’s Hospital, The Second Affiliated Hospital Daishan Branch, Zhejiang University School of Medicine, Zhoushan, China; 4 Department of Critical Care Medicine, Zhejiang Qingyuan People’s Hospital, The Second Affiliated Hospital Qingyuan Branch, Zhejiang University School of Medicine, Lishui, China

**Keywords:** Mechanical ventilation, Tracheostomy, Optimal timing, Intensive care

## Abstract

**Objectives:**

The timing of tracheostomy for critically ill patients on mechanical ventilation (MV) is a topic of controversy. Our objective was to determine the most suitable timing for tracheostomy in patients undergoing MV.

**Design:**

Retrospective cohort study.

**Setting and participants:**

One thousand eight hundred eighty-four hospitalisations received tracheostomy from January 2011 to December 2020 in a Chinese tertiary hospital.

**Methods:**

Tracheostomy timing was divided into three groups: early tracheostomy (ET), intermediate tracheostomy (IMT), and late tracheostomy (LT), based on the duration from tracheal intubation to tracheostomy. We established two criteria to classify the timing of tracheostomy for data analysis: Criteria I (ET ≤ 5 days, 5 days < IMT ≤ 10 days, LT > 10 days) and Criteria II (ET ≤ 7 days, 7 days < IMT ≤ 14 days, LT > 14 days). Parameters such as length of ICU stay, length of hospital stay, and duration of MV were used to evaluate outcomes. Additionally, the outcomes were categorized as good prognosis, poor prognosis, and death based on the manner of hospital discharge. Student’s t-test, analysis of variance (ANOVA), Mann–Whitney U test, Kruskal–Wallis test, Chi-square test, and Fisher’s exact test were employed as appropriate to assess differences in demographic data and individual characteristics among the ET, IMT, and LT groups. Univariate Cox regression model and multivariable Cox proportional hazards regression model were utilized to determine whether delaying tracheostomy would increase the risk of death.

**Results:**

In both of two criterion, patients with delayed tracheostomies had longer hospital stays (*p* < 0.001), ICU stays (*p* < 0.001), total time receiving MV (*p* < 0.001), time receiving MV before tracheostomy (*p* < 0.001), time receiving MV after tracheostomy (*p* < 0.001), and sedation durations. Similar results were also found in sub-population diagnosed as trauma, neurogenic or digestive disorders. Multinomial Logistic regression identified LT was independently associated with poor prognosis, whereas ET conferred no clinical benefits compared with IMT.

**Conclusions:**

In a mixed ICU population, delayed tracheostomy prolonged ICU and hospital stays, sedation durations, and time receiving MV. Multinomial logistic regression analysis identified delayed tracheostomies as independently correlated with worse outcomes.

**Trial registration:**

ChiCTR2100043905. Registered 05 March 2021.http://www.chictr.org.cn/listbycreater.aspx

**Supplementary Information:**

The online version contains supplementary material available at 10.1186/s12871-024-02411-1.

## Background

Critically ill patients with various ectiology such as pneumonia, trauma, central injury, infection and acute respiratory syndrome distress (ARDS), would present with hypoxia, carbon dioxide storage, and respiratory failure sometimes. For those patients, mechanical ventilation (MV) support is commonly used to help them get through the dangerous period. Endotracheal intubation is the most convenient method initiating MV but comes with its disadvantages including poorly tolerated by awake patients, potentially disastrous dislodgement, inconvenient secretion drainage, and its interference with oral care, feeding, and communication [1]. More and more patients receiving long-term MV are treated with tracheostomy [2, 3]. Tracheostomy can prevent more damage to the mouth and larynx, reduce airway resistance, decrease the risk of ventilator-associated pneumonia (VAP), improve patient comfort, decrease sedative administration, and enhance patient’s mobility and ability to eat orally, which facilitates patients weaning off and being discharged from the intensive care unit (ICU) as early as possible [4–7]. However, tracheostomy is an invasive procedure that carries the risk of developing complications, such as hemorrhage, infection at the incision site, obstruction of the tube, accidental removal of the tube, formation of granulation tissue, tracheal stenosis, tracheomalacia, and more [8, 9]. Physicians have historically judged the timing of tracheostomy by weighing the benefits and risks. However, a growing of literatures showed multidisciplinary, team-based approaches for planning tracheostomy and coordinating postoperative care could reduce adverse events and improve prognosis [10–12]. Most patients with ARDS receive a tracheostomy beyond 7 days after symptom onset, whereas nearly half of the tracheostomies were performed within 7 days post-intubation in mechanically-ventilated trauma patients [13, 14]. Furthermore, there was great variation between hospitals regarding the timing of tracheostomy [13]. Significant association between decreased in-hospital mortality and early tracheostomy (ET < post-operation 10 days) were found in patients with MV in cardiac surgery [15]. Gillis A et al. also reported that late tracheostomy (LT > 10 days post-intubation) had higher mortality (19% vs 13% p < 0.01) in a trauma center [16]. However, other studies also investigating patients with traumatic injuries didn’t observe significantly decreased mortality among patients undergoing ET [17–19]. The definition of the timing of tracheostomy (ET and LT) varies among the studies mentioned above. Early tracheostomy was performed less than 8 days post-intubation in the study peformed by Alali AS et al. [17], whereas tracheostomy performed less than 4 days post-intubation was defined as early tracheostomy in a research conducted by Anand T et al. [18]. The conclusions originating from several meta-analyses investigating the effects of ET in decreasing mortality were also inconsistent [20–22]. Recently, a multicenter cohort study revealed that delayed tracheostomy (> 7d after ICU admission) was independently associated with increased in-hospital mortality [23]. Some studies showed performing ET could reduce length of ICU stay for patients with MV [15–17, 21, 22, 24–26], but the other negated this correlation [27]. Furthermore, whether delayed tracheostomy would prolong the duration of MV and increase the incidence of VAP also remains controversial [18, 21, 22, 24, 25, 28]. Briefly, previous studies reported divergent results concerning the relationship between tracheostomy timing and clinical outcome. Besides, the definitions of early tracheostomy (ET) and later tracheostomy (LT) varied in different studies, complicating the interpretation of the results. Furthermore, many studies were performed in a certain category of patients, and the conclusions were often limited by insufficient sample size. To clarify the optimal timing of a tracheostomy for critically ill patients, we retrospectively analyzed the clinical data of patients treated with a tracheostomy over the past decade and compared the clinical outcomes of patients with different tracheostomy timings.

## Materials and methods

### Study design and participants

We conducted a retrospective observational study at the Second Affiliated Hospital, Zhejiang University School of Medicine. Patients who were admitted to all of the ICUs from January 2011 to December 2020 and treated with MV were eligible. The trial was approved by the hospital’s ethics committee of the Second Affiliated Hospital, Zhejiang University School of Medicine (2021 Ethical Review No. 0210) and registered in the Chinese Clinical Trial Registry (ChiCTR2100043905). Two researchers extracted data on the included patients from the electronic medical system independently. A third researcher would randomly extract partial data for manual verification to ensure the accuracy of data. Patients aged 18 years and older with a database record of tracheostomy events were included. Patients were excluded if they met any of the following criteria: emergency tracheostomy without prior tracheal intubation, uncertain time of tracheal intubation, weaning from intubation successfully before tracheostomy (tracheal intubation removed for more than 48 h), non-first-time hospitalized patients, without documented tracheostomy method, or without MV.

### Definitions

Tracheostomy timing was categorized into early tracheostomy (ET), intermediate tracheostomy (IMT), and late tracheostomy (LT) groups according to the duration from tracheal intubation to tracheostomy. We established two criteria to categorize the timing of tracheostomy in order to analyze the data comprehensively: Criteria I (ET ≤ 5 days, 5 days < IMT ≤ 10 days, LT > 10 days) and Criteria II (ET ≤ 7 days, 7 days < IMT ≤ 14 days, LT > 14 days).Baseline characteristics included age, sex, the main reason for hospital admission (coma, neurogenic, respiratory, cardiovascular, or neuromuscular injury, digestive disorder, trauma, and more), type of tracheostomy (surgical or percutaneous). The date of the tracheostomy, tracheal intubation, MV, sedation, and hospital and ICU admissions were also recorded in the electronic case system, based on which tracheostomy timing characteristic could be analyzed. Important parameters for evaluating the timing of tracheostomy and prognosis in patients include the duration of ICU stay, duration of hospital stay, length of MV, and duration of sedation. In this study, patients’ primary outcomes were categorized as good prognosis, poor prognosis, or death based on their method of hospital discharge. Patients who met the discharge criteria through assessment by attending physicians and were permitted to be discharged or transferred to a rehabilitation hospital were considered to have a good prognosis. However, in some cases, patients’ condition continued to deteriorate and reached a terminal stage. Further medical intervention was deemed futile for these patients, as it only increased their suffering and medical costs. In such situations, clinicians would inform the patients’ family members about their condition and poor prognosis. Consequently, some families decided to discharge the patients home, where they could spend their remaining time in the comfort of their own surroundings. Although these patients were still alive upon leaving the hospital, their impending death made their prognosis poor.

### Statistical analysis

The continuous variables are described as means ± SD if distributed normally, and otherwise shown as medians (Interquartile range, IQR). The categorical variables are presented as counts and percentages. Student’s t-test, analysis of variance (ANOVA), Mann–Whitney U test, Kruskal–Wallis test, Chi-square test, and Fisher’s exact test were used as appropriate to test for differences in demographic data and individual characteristics among ET, IMT, and LT. Variables with *P* < 0.10 identified by the univariate analysis or those were considered clinically to affect outcomes for patients, were included for further multivariable analysis. As for independent variable, the timing of tracheostomy is categorical variable after grouping, in which IMT is set as the reference category. The factors that might affect mortality, including age, type of tracheotomy, mode of administration, and tracheostomy time, etc. were analyzed using a univariate Cox regression model. Then, variables with *P* < 0.10 identified by the univariate analysis or clinically correlated with mortality were included in a multivariable Cox proportional hazards regression model. Multivariable Cox proportional hazards regression model was achieved, as the proportional risk model was satisfied (*P* > 0.05). The outcome was categorized as good prognosis, poor prognosis and death according to the way of hospital discharge, which was the dependent variable and an ordered multicategorical variable. Multinomial Logistic regression was achieved, as there is no multicollinearity between independent variables and the proportionality advantage assumption was satisfied, which is designed to investigate whether delayed tracheostomy would apply a negative influence on patients. The goodness of fit was tested for all logistic regression and Cox regression models. All tests were two-tailed and statistical significance was determined at an α level of 0.05. Statistical analyses were performed using R and SPSS 20.0.

## Results

### Overview and baseline characteristics

**Fig. 1:**
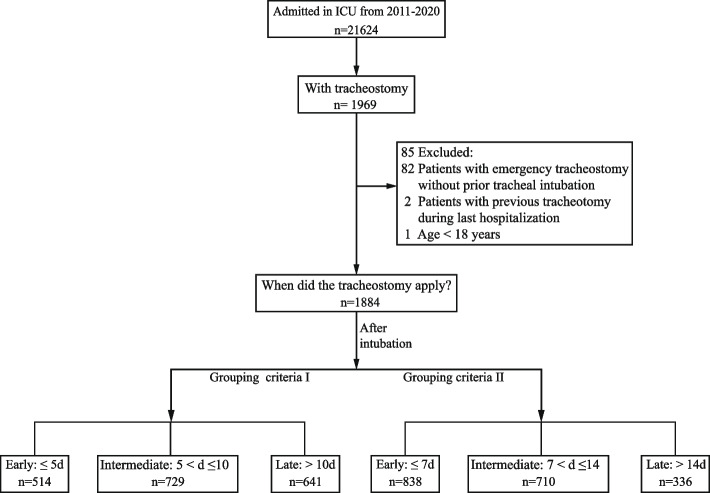
Flow diagram of specific process of the trial, illustrating the number of patients in each step and each group. Grouping criteria I: ET (≤ 5d), IMT (5 < d ≤ 10), LT (> 10d). Grouping criteria I: ET (≤ 7d), IMT (7 < d ≤ 14), LT (> 14d). ET = early tracheostomy, IMT = intermediate tracheostomy, LT = late tracheostomy

A total of 21,624 patients were admitted to the ICU of our hospital, of whom 1,969 (9.1%) underwent a tracheostomy. 85 patients were excluded, among whom 82 were applied emergency tracheostomy directly (without tracheal intubation before tracheostomy), two had a previous history tracheostomy, one patient was younger than 18 years old (Fig. 1). The remaining 1,884 patients included in the present study had a median (IQR) age of 65 (55–73) and 68.5% were male. The primary diagnoses included neurogenic injury (66.1%) followed by trauma (16.3%) (Supplemental Table 1). We found most patients underwent a tracheostomy within 14 days, and the timing distribution is depicted in detail in Supplemental Fig. 1*.* These patients were divided into ET, IMT, and LT groups according to the timing of their tracheostomy, as illustrated in Fig. 1. The baseline characteristics of the cohort are presented in Supplemental Table 1 according to group (ET, IMT, LT).

### Patients with delayed tracheostomies had longer duration of ICU stays and time receiving MV

**Fig. 2:**
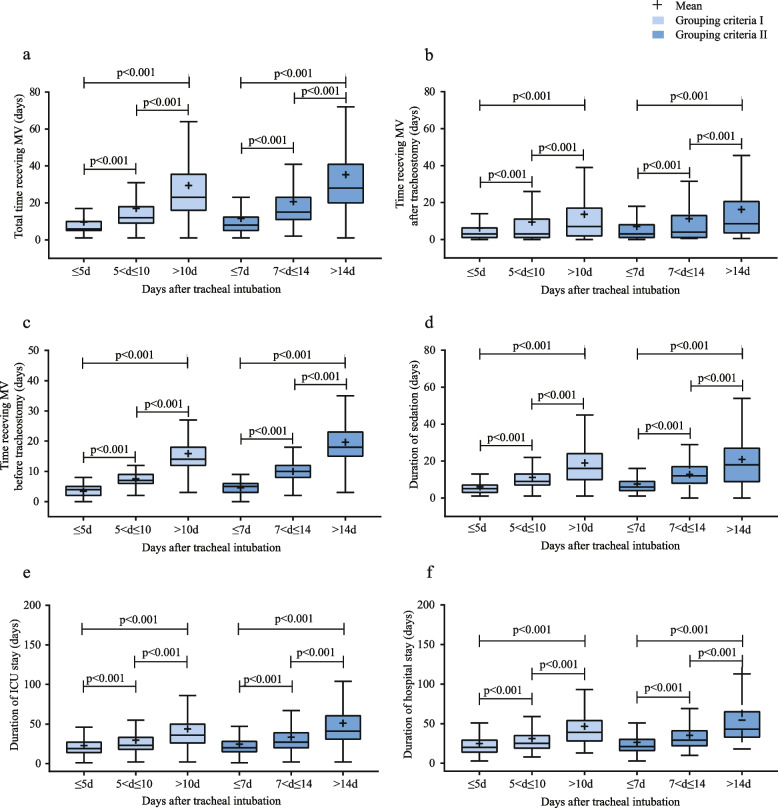
Compare secondary outcomes between ET, IMT, and LT. **a** total MV time, **b** MV time after tracheostomy, **c** time receiving tracheostomy before tracheostomy, **d** duration of sedation, **e** duration of ICU stays, **f** duration of hospital stays. Boxes represent IQR, center lines denote the median and symbol plus (+) denote mean. The upper whisker line represents the smaller value between the maximum in the data set and 75th percentile plus 1.5 times IQR. Grouping criteria I: ET (≤ 5d), IMT (5 < d ≤ 10), LT (> 10d). Grouping criteria II: ET (≤ 7d), IMT (7 < d ≤ 14), LT (> 14d). The lower whisker line represents the greater value between the minimum and the 25th percentile minus 1.5 IQR. n = number, IQR = interquartile range, MV = mechanical ventilation, ICU = intensive care unit, ET = early tracheostomy, IMT = intermediate tracheostomy, LT = late tracheostomy

Among the 1,884 patients, 1,589 had good prognosis, 249 had poor prognosis, and 46 died upon leaving the hospital. Based on the grouping criteria I (LT: > 10d vs IMT: 5 < d ≤ 10 vs ET: ≤ 5d), the patients with delayed tracheostomies had longer hospital stays (median: 39 days (IQR: 28–54) vs 25 days (19–35) vs 20 days (14–29), *p* < 0.001), ICU stays (median: 36 days (IQR:26–50) vs 23 days (18–33) vs 19 days (14–27), *p* < 0.001), total time receiving MV (median:23 days (IQR:16–35.5) vs 12 days (IQR: 9–18) vs 6 days (IQR: 5–10), *p* < 0.001), time receiving MV before tracheostomy (median:14 days (IQR: 12–18) vs 7 days (IQR: 6–9) vs 4 days (IQR: 2–5), *p* < 0.001), time receiving MV after tracheostomy (median: 7 days (IQR: 2–17) vs 3 days (IQR: 1–11) vs 3 days (IQR: 1–7), *p* < 0.001), and sedation durations (median: 16 days (IQR: 10–24) vs 9 days (IQR: 7–13) vs 5 days (IQR: 3–7), *p* < 0.001) (Fig. 2, Table 1). Dividing the patients according to grouping criteria II (LT: > 14d vs IMT: 7 < d ≤ 14 vs ET: ≤ 7d, *p* < 0.001) yielded same results (Table 2). Patients with delayed tracheostomies (LT > 10d vs IMT: 5 < d ≤ 10 vs ET < 5d) had higher in-hospital mortality rates (4.7% vs 1.5% vs 1.0%), and have lower percentage of good prognosis upon discharge (78.8% vs 85.9% vs 89.1%) (Table 1). We found similar results when performing an analysis based on grouping criteria II (ET ≤ 7d, IMT: 7 < d ≤ 14, LT > 14d) (Table 2).

**Table 1:** Comparison of outcomes between ET, IMT and LT in sub-populationof subjects

The timing of tracheostomy after intubation	Discharge Status, %	Length of stay, mean (IQR)		Time receiving MV, mean (IQR)		
	Death/Poor/Good	In-hospital	ICU	Total	Before TT	After TT
**Grouping Criteria I (ET: ≤ 5d, IMT: 5 < d ≤ 10, LT: > 10d)**						
**Included patients, *n* = 1884**						
ET (*n* = 514)	1.0 / 9.9 / 89.1	20(14–29)	19(14–27)	6(5–10)	4(2–5)	3(1–7)
IMT (*n* = 729)	1.5 / 12.6 / 85.9	25(19–35)	23(18–33)	12(9–18)	7(6–9)	3(1–11)
LT (*n* = 641)	4.7 / 16.5 / 78.8	39(28–54)	36(26–50)	23(16–35.5)	14(12–18)	7(2–17)
*p*-value	< 0.05	< 0.001	< 0.001	< 0.001	< 0.001	< 0.001
**Sub-population analysis**						
Neurogenic injury, *n* = 1246						
ET (*n* = 359)	1.4 / 8.9 / 89.7	19(14–27)	18(14–25)	6(5–10)	4(2–5)	3(1–6)
IMT (*n* = 509)	1.0 / 9.4 / 89.6	24(19–33)	23(18–31)	11(9–18)	8(6–9)	3(1–10)
LT (*n* = 378)	1.1 / 14 / 84.9	35(26–48)	32.5(25–45)	20(15–32)	14(11–17)	5(2–14)
Trauma, *n* = 308						
ET (*n* = 82)	0 / 8.5 / 91.5	21(17–31)	21(13–29)	7(5–10)	4(3–5)	3(1–6)
IMT (*n* = 139)	1.4 / 16.5 / 82	24(19–34)	23(17–34)	9(11–16)	7(6–8)	3(1–8)
LT (*n* = 87)	4.6 / 18.4 / 77	38(28–48)	38(28–47)	21(17–29)	13(11–17)	7(3–16)
Respiratory disease, *n* = 85						
ET (*n* = 13)	0 / 38.5 / 61.5	39(13–55)	34(12–55)	13(5–36.5)	3(1.5–4)	9(1.5–33)
IMT (*n* = 14)	14.3 / 42.9 / 42.9	32(20–45.5)	27(19.5–36)	19(12–32)	7.5(7–9)	11.5(4–23)
LT (*n* = 58)	15.5 / 24.1 / 60.3	48(35–74.5)	44.5(31–71)	29(19–46)	16(12–21)	11(5–29)
Cardiovascular disease, *n* = 51						
ET (*n* = 16)	0 / 18.8 / 81.3	21.5(15.5–29)	20.5(15.5–28)	8.5(5–13)	4(3–5)	4(2–9)
IMT (*n* = 16)	6.3 / 25 / 68.8	35(22.5–58)	26(18–58)	21.5(9–35)	7(7–9)	13.5(2–26.5)
LT (*n* = 19)	10.5 / 10.5 / 78.9	49(42–57)	42(26–57)	25(18–37)	13(12–22)	12(3–19)
Coma, *n* = 28						
ET (*n* = 11)	0 / 0/ 100	29(17–40)	29(17–37)	29(17–40)	4(3–5)	2(1–6)
IMT (*n* = 9)	0 / 44.4 / 55.6	38(24–65.5)	38(24–64.5)	13(10.5–32)	9(6.5–9.5)	7(2.5–23)
LT (*n* = 8)	0 / 12.5 / 87.5	44(37–63.5)	44(37–63)	33.5(27–45)	14.5(11–23.5)	16(10–25)
Neuromuscular disease, *n* = 33						
ET (*n* = 4)	0 / 25 / 75	39(16.5–70)	39(14–70)	22(10–58)	4.5(4–5)	17.5(5–54)
IMT (*n* = 7)	0 / 14.3 / 85.7	47(22–63)	36(22–63)	19(6–58)	8(6–9)	12(2–48)
LT (*n* = 22)	4.5 / 22.7 / 72.7	56(42–72.5)	53(37.5–72.5)	35.5(22–64)	14.5(12–21.5)	17(9–36.5)
Digestive disorder, *n* = 53						
ET (*n* = 4)	0 / 50 / 50	24(14–44)	18.5(10–26)	10(4–29)	3(0.5–5)	5.5(1–28)
IMT (*n* = 14)	0 / 35.7 / 64.3	27.5(21.5–36)	24(19–31)	15(10–21.5)	7.5(6–8)	9(2–14)
LT (*n* = 35)	25.7 / 20 / 54.3	47(35–70)	42(29–66)	30(21–45)	16(13–22)	12(3–21)
Others, *n* = 80						
ET (*n* = 25)	0 / 4.0 / 96	26(15.5–41)	20(13.5–34.5)	5(3–9)	3(1–4)	2(0–5)
IMT (*n* = 21)	4.8 / 4.8 / 90.5	35(26–56.5)	33(21–49.5)	12(9.5–29.5)	7(6–9)	4(1–21)
LT (*n* = 34)	2.9 / 23.5 / 73.5	40.5(33–65)	39(27–62.5)	25.5(17–37)	15.5(12–22)	6.5(2–20)

The timing of tracheostomy was measured by grouping criteria I (ET ≤ 5d; 5d < IMT ≤ 10d; LT > 10d). *y* year, *n* number, *IQR* Interquartile Range, *d* day, *TT* tracheostomy

**Table 2:** Comparison of outcomes between ET, IMT and LT by grouping criteria II (ET ≤ 7d; 7d < IMT ≤ 14d; LT > 14d)

The timing of tracheostomy after intubation	Discharge Status, %	Length of stay, median (IQR)		Time receiving MV, median (IQR)		
	Death/Poor/Good	In-hospital	ICU	Total	Before TT	After TT
**Grouping Criteria II (ET: ≤ 7d, IMT: 7 < d ≤ 14, LT: > 14d)**						
Included patients, *n* = 1884						
ET (*n* = 838)	1.4 / 10.7 / 87.8	21(16–30)	20(15–28)	8(5–12.25)	5(3–6)	3(1–8)
IMT (*n* = 710)	1.5 / 14.9 / 83.5	29(22–41)	27(20–39)	15(11–23)	10(8–12)	4(1–13)
LT (*n* = 336)	6.8 / 15.8 / 77.4	43(33–65)	41(31–60.5)	28(20–41)	18(15–23)	9(3–20)
*P*-value	< 0.05	< 0.001	< 0.001	< 0.001	< 0.001	< 0.001
Sub-population analysis						
Neurogenic injury, *n* = 1246						
ET (*n* = 579)	1.6 / 9.5 / 88.9	21(16–28)	19(15–17)	8(5–12)	5(3–6)	3(1–7)
IMT (*n* = 475)	0.4 / 11.2 / 88.4	28(21–37)	26(20–35)	14(11–21)	10(8–11)	4(1–11)
LT (*n* = 192)	1.6 / 13 / 85.4	40(29–57)	25(18–38)	25(18–38)	17(15–21)	7(2–15)
Trauma, *n* = 308						
ET (*n* = 148)	0.7 / 7.4 / 91.9	22(17–32)	21(15–31)	8(6–12)	5(4–6)	3(1–6.75)
IMT (*n* = 126)	1.6 / 23.8 / 74.6	27(21–40)	27(19–40)	16(11–21)	9(8–11)	4.5(2–11)
LT (*n* = 34)	8.8 / 14.7 / 76.5	40(31–56)	40(30–56)	28(19–40.5)	18.75(15–22.5)	6.5(2–20)
Respiratory disease, *n* = 85						
ET (*n* = 20)	5.0 / 45 / 50	30.5(13–48.5)	26(13–48)	14(6–30)	4(2–6)	9.5(3–23)
IMT (*n* = 26)	11.5 / 30.8 / 57.7	44.5(31–60)	41(23–51)	22.5(16.5–41)	12(9–13)	11.5(5–29.5)
LT (*n* = 39)	17.9 / 20.5 / 61.5	56(36–81)	50(33–73)	31(22–51)	19(16–26)	11(5–29)
Cardiovascular disease, *n* = 51						
ET (*n* = 26)	0 / 26.9 / 73.1	24(18–35)	21(16.5–28)	9.5(7–22)	5(4–7)	4.5(2–15)
IMT (*n* = 13)	7.7 / 7.7 / 84.6	49(25–58)	28(23–57)	18(12.5–33)	9(9–12)	12(1–22.5)
LT (*n* = 12)	16.7 / 8.3 / 75	54.5(43.5–73)	44(35–58)	36(23–48)	17.5(13–26.5)	13.5(10–22.5)
Coma, *n* = 28						
ET (*n* = 15)	0 / 13.3 / 86.7	29(18–34)	29(18–34)	8(5–13)	5(3–6)	3(1–7)
IMT (*n* = 8)	0 / 25 / 75	42.5(38–68)	42.5(38–66)	31.5(15.5–37)	10(9–11)	20.5(5–26)
LT (*n* = 5)	0 / 20 / 80	56(39–79.5)	56(39–79)	35(23.5–58)	16(14–28)	14(5–37)
Neuromuscular disease, *n* = 33						
ET (*n* = 7)	0 / 28.6 / 71.4	27(13–51)	27(10–51)	10(6–34)	5(4–6)	5(2–30)
IMT (*n* = 15)	0 / 20 / 80	50(35–59)	50(26–58)	25(18–53)	11(10–12)	14(6–41)
LT (*n* = 11)	9.1 / 18.2 / 72.7	71(64–82)	67(53–81)	53(32–71)	21(18–27)	21(14–41)
Digestive disorder, *n* = 53						
ET (*n* = 9)	0 / 33.3 / 66.7	28(20–39.5)	23(15.5–27.5)	14(6.5–16)	6(3–6)	9(1–10.5)
IMT (*n* = 19)	15.8 / 26.3 / 57.9	30(22–45)	25(21–41)	20(13–26)	9(8–12)	10(2–16)
LT (*n* = 25)	24 / 24 / 52	51(40.5–79.5)	43(32–73)	32(24.5–44.5)	18(15.5–23)	13(5–20)
Others, *n* = 80						
ET (*n* = 34)	2.9 / 2.9 / 94.1	29.5(19–41)	23.5(17.5–35)	6(4–10)	4(1–5)	2(1–5)
IMT (*n* = 28)	0 / 14.3 / 85.7	35.5(27–57.5)	30(21–52)	18.5(11–26)	10(8–12)	7.5(1–15)
LT (*n* = 18)	5.6 / 27.8 / 66.7	52.5(38–66)	44.5(36.5–64)	34.5(25–38)	21.5(18.5–27.5)	9.5(3–20.5)

### Delayed tracheostomies prolonged duration of ICU stays and time receiving MV in sub-population with trauma, neurogenic injury or digestive disorder

We categorized patients according to their reasons for hospital admission and performed a stratified analysis among these etiology-based sub-populations. Based on grouping criteria I (LT: > 10d vs IMT: 5 < d ≤ 10 vs ET: ≤ 5d), within the populations with trauma (*n* = 308), neurogenic injury (*n* = 1246), and digestive disorder (*n* = 53), patients with delayed tracheostomy had significantly prolonged ICU and hospital stays, total MV duration, and MV duration before tracheostomy (Table 1). For patients with cardiovascular disease (*n* = 51) and those in a coma (*n* = 28), a delayed tracheostomy prolonged the duration of their hospital and ICU stay and MV duration before tracheostomy, respectively, but there was no significant inter-group difference in MV duration after tracheostomy. LT (> 10d) was correlated significantly with prolonged MV duration, ICU and hospital stays in ICU patients with respiratory (*n* = 85) and neuromuscular diseases (*n* = 33), however, the differences between the ET and IMT groups didn’t reach statistical significance (Table 1). Sub-population analysis according to etiology based on grouping criteria II was depicted in Table 2.

### Patients with late tracheostomies have a poorer prognosis upon discharge by multinomial logistic regression analysis

We performed a multinomial logistic regression analysis with patient outcome as the dependent variable. These outcomes were grouped as good prognosis, poor prognosis and death according to the way of hospital discharge. With IMT (5 < d ≤ 10d) as a reference category, LT (> 10d after tracheal intubation), was independently associated with poor prognosis (RR = 1.6, 95% CI = 1.07–2.38, *p* < 0.001) after adjusting confounding factors such as age, sex, type of tracheostomy, total MV, duration of ICU stays and MV after tracheostomy (Table 3). Consistently, LT (> 10d after ICU admission) was independently associated with poor prognosis (RR = 2, 95% CI = 1.34–2.97, *p* < 0.01, Supplemental Table 2). However, there was no significant difference in patient prognosis (poor vs good) between ET and IMT, regardless of whether the timing of the tracheostomy was measured by the duration after tracheal intubation (ET ≤ 5d vs IMT: 5 < d ≤ 10, p = 0.2; ET ≤ 7d vs IMT: 7 < d ≤ 14, *p* = 0.09) or ICU admission (*p* = 0.10, *p* = 0.03).

**Table 3:** Multinomial logistic regression analysis with outcomes at discharge as dependent variable

Outcome	Poor prognosis		Death	
	RR(95%CI)^**a1**^	RR2(95%CI)^**b1**^	RR(95%CI)^**a2**^	RR(95%CI)^**b2**^
The timing of tracheostomy (from intubation, days)				
Grouping criteria I				
≤ 5d	0.78(0.52,1.16)	NA	0.79(0.25,2.46)	NA
5 < d ≤ 10d	Reference	NA	Reference	NA
> 10d	1.6(1.07,2.38) *	NA	1.56(0.66,3.7)	NA
Grouping criteria II				
≤ 7d	NA	0.73(0.51,1.05)	NA	1.42(0.58,3.51)
7 < d ≤ 14	NA	Reference	NA	Reference
> 14d	NA	1.2(0.73,1.96)	NA	2.04(0.79,5.31)
Age, y				
≤ 50	Reference			
50–60	0.82(0.52,1.28)	0.82(0.52,1.29)	2.5(0.5,12.7)	2.57(0.51,12.98)
60–70	0.91(0.59,1.39)	0.92(0.6,1.41)	1.4(0.25,7.6)	1.43(0.26,7.95)
70–80	1.16(0.75,1.79)	1.16(0.75,1.78)	2.9(0.59,14.2)	2.88(0.58,14.2)
> 80	0.86(0.5,1.47)	0.85(0.5,1.46)	11.7(2.7,51.6)	11.93(2.7,52.72) **
Sex				
Female	Reference			
Male	1.17(0.86,1.58)	1.16(0.85,1.57)	1.8(0.82,3.88)	1.75(0.8,3.82)
Duration of stay				
hospital	1.02(1,1.05)	1.02(1,1.05)	1.04(1.01,1.07)*	1.04(1,1.07)*
ICU	0.93(0.9,0.95)***	0.93(0.9,0.95)***	0.93(0.89,0.97)***	0.93(0.9,0.97)***
Type of tracheostomy				
Percutaneous	Reference			
Surgical	1.38(1.04,1.84) *	1.37(1.03,1.82) *	2.07(1.07,3.98) *	2.14(1.1,4.16)*
Length of MV				
Total	1.03(0.99,1.06)	1.04(1,1.08) *	1.06(1.01,1.11) *	1.06(1.01,1.12)*
after TT	1.04(1.01,1.08) *	1.03(1,1.07)	0.98(0.94,1.03) *	0.98(0.94,1.03)
Length of sedation	1.01(1,1.02)	1.01(1,1.03) *	1.02(1,1.04)	1.02(1.01,1.04)*

The timing of tracheostomy was measured by duration after tracheal intubation. Multinomial logistic regression analysis with outcomes (good prognosis, poor prognosis and death) at discharge as dependent variable. The outcomes includes good prognosis, poor prognosis and death, in which good prognosis was the reference. RR^**a1**^ indicate the risk ratio of poor prognosis, whereas RR^**b1**^ suggest the risk ratio of death (the timing of tracheostomy was grouped by grouping criteria I). RR^**a2**^ indicate the risk ratio of poor prognosis, whereas RR^**b2**^ suggest the risk ratio of death (the timing of tracheostomy was grouped by grouping criteria II). *Y* year, *d* day, w week, ICU Intensive care unit, *MV* mechanical ventilation, *RR* risk ratio, *CI* Confidence Interval, *NA* not available

*P <0.05; **P <0.01; ***P <0.001

### No significant difference was found in hazard ratio of death for patients with different timing of tracheostomies

To assess the risk of death, we not only performed multinomial logistic regression analysis, but also conducted univariate and multivariate Cox hazard analyses of related factors. Regarding IMT as a reference, no significant difference in risk of death was found in patients with late tracheostomies (Table 4 and Supplemental Table 2). Interestingly, we found patients with ET (≤ 5d after ICU admission) have lower HR of death (HR_3_ = 0.26, 95%CI = 0.08–0.89, *p* = 0.03, Table 4). However, no significant difference in HR of death were found in other grouping criteria, whether the timing of tracheostomy were measured by duration after intubation or ICU admission.

**Table 4:** Multivariate Cox hazard analysis for patients treated by tracheostomy

The timing of tracheostomy	From intubation to tracheostomy			From ICU admission to tracheostomy		
	HR_**1**_(95%CI)		HR_**2**_(95%CI)	HR_**3**_(95%CI)		HR_**4**_(95%CI)
Grouping criteria I						
≤ 5d	0.53(0.17,1.70)	NA		0.26(0.08,0.89)*	NA	
5 < d ≤ 10	Reference	NA		Reference	NA	
> 10d	0.86(0.37,1.96)	NA		1.33(0.56,3.14)	NA	
Grouping criteria II						
≤ 7d	NA	1.3( 0.54,3.31)		NA	0.91(0.36,2.34)	
7 < d ≤ 14	NA	Reference		NA	Reference	
> 14d	NA	0.89(0.36,2.19)		NA	1.67(0.65,4.31)	
Age, y						
≤ 50	Reference					
50–60	4.14(0.63,27.3)	4.32 (0.66, 28.4)		3.4(0.55,21.2)	3.96(0.58,26.8)	
60–70	1.67(0.23,12.1)	1.72( 0.24, 12.5)		1.51(0.22,10.4)	1.70(0.23,12.6)	
70–80	2.33(0.34,15.9)	2.55(0.38,17.1)		2.12(0.33,13.6)	2.40(0.34,16.7)	
> 80	11.7(1.97,69.8) **	11.3( 1.89,67.0) **		9.54(1.69,53.8)*	11.0(1.80,67.5)**	
Sex						
Female	Reference					
Male	1.30(0.61,2.80)	1.26( 0.59, 2.71)		1.20(0.56,2.58)	1.18(0.55,2.53)	
Type of tracheostomy						
Percutaneous	Reference					
Surgical	1.30(0.67,2.51)	1.49(0.77,2.90)		1.32(0.70,2.51)	1.29(0.68,2.45)	
Length of MV						
Total	1.05(1.01–1.10) *	1.07( 1.02,1.11)**		1.05(1.00,1.09)*	1.04(0.99,1.09)	
After tracheostomy	0.94(0.91,0.98) **	0.93(0.90,0.97)**		0.96(0.92,0.99)*	0.96(0.91,0.99)*	
Length of sedation						
	1.02(0.99,1.03)	1.02(1.0,1.04)		1.01(0.99,1.03)	1.01(0.99,1.03)	
Duration of ICU stay	0.91(0.89,0.93) ***	0.91( 0.89,0.93) ***		0.89(0.87,0.92) ***	0.91(0.88,0.93) ***	

Multivariate Cox hazard analysis for ICU patients with tracheostomy. The timing of tracheostomy was measured duration after tracheal intubation. In cox regression analysis, the event was the death occurring before leaving the hospital and the observation time was the duration of hospital stay. HR_**1**_, HR_**2**_, HR_**3**_, HR_**4**_ were the hazard ratio of death in cox regression models, in which the independent variable the timing of tracheostomy was grouped by different criteria. *Y* year, *d* day, *w* week, *HR* Hazard Ratio, *ICU* Intensive care unit, *CI* Confidence Interval, *MV* mechanical ventilation, *NA* not available

*P <0.05; **P <0.01; ***P <0.001

## Discussion

In order to figure out an appropriate timing of tracheostomy, we retrospectively analyzed 1,884 critically ill patients who underwent tracheostomies. In univariate analyses, patients with delayed tracheostomies had higher in-hospital and ICU mortality rates, a higher percentage of poor prognosis during discharge, longer hospital and ICU stays, longer duration of MV (whether before or after the tracheostomy), and increased sedation duration. These differences were statistically significant in mixed ICU population and sub-populations diagnosed as neurogenic injury, trauma, and digestive disorders. Multinomial logistic regression analysis identified LT as independently correlated with worse outcomes. In summary, tracheostomy should be performed within 10 days post-intubation for patents who have a high possibility of long-term MV. It worsened patients’ outcomes, prolonged the duration of ICU and hospital stay, and increased MV duration when the tracheostomy was applied too late. It inevitably requires more medical resources, causes patients discomfort during hospitalization, and affects their rehabilitation after discharge.

Yet, no significant difference in mortality risk was found among patients with different tracheostomy timings in our multivariable Cox regression analysis, similar to findings in previous studies with various definitions of LT and diverse populations of subjects [17, 18, 22, 27, 29]. We observed that patients who underwent late tracheostomy had a higher mortality rate (1.0% *VS* 1.5% *VS* 4.7%). However, there was no statistically significant increase in the risk of death for these patients. The findings regarding mortality and the risk of mortality in our study are inconclusive. This could be attributed to the limited number of deaths in our sample, which may have introduced some bias. Additionally, the risk of mortality analysis was adjusted for confounding factors using algorithmic correction. In fact, death is an extreme outcome, thus it is inappropriate to deny benefits by solely comparing mortality [30, 31]. In the present study, we innovatively categorized patients into good prognosis, poor prognosis and death according to the way of hospital discharge. In clinical practice, spending remaining life with family at home is a fairly common choice for patients with terminal stage. However, it is difficult to get the specific reasons of early discharge for each patient for now due to the retrospective nature of our study. We didn’t know whether financial concern of prolonged hospitalization is also a motivation of early discharge or not, and how many patients were influenced. Besides, even for patients who discharged following physicians’ advices, rehabilitation is also a long process full of grim survivorship challenges such as cognition, weakness, mental health, speech, swallowing, and breathing [32, 33]. Thus, it is critical to perform long-term follow-up and design detailed criteria to standardize evaluation of patients’ rehabilitation in future investigation.

Stacey L et al. reported that the intraoperative, early (< 1 week), and late complication rates were 1.4%, 5.6%, and 7.1%, while postoperative bleeding (2.6%) and airway stenosis (1.7%) was identified as the most common early and late complication, respectively [8]. Actually, tracheostomy is an operation with adequate safety due to the low rate of complication. In our study, delaying a tracheostomy until after 10 days post-intubation (RR = 1.6, 95%CI = 1.07–2.38, *p* < 0.001) was revealed to correlate independently with a poor prognosis. Consistent results were found when we use duration from ICU admission to measure the timing of tracheostomy (RR = 2, 95%CI = 1.34–2.97, *p* < 0.01). Interestingly, no similar benefit was found when shifting the timing of the tracheostomy earlier (< 5 days). Physicians should evaluate patients within the first week post-intubation and decide whether or not to perform a tracheostomy within 10 days post-intubation. Contrary to our findings, Siempos et al. reported a lower all-cause mortality rate in patients with ET (OR = 0.72, 95% CI = 0.53–0.98, *p* = 0.04) in their analysis of studies published between 1984 and 2013 [20]. The incidence of tracheostomies increased, while in-hospital mortality declined (38.1% vs 14.7%, *p* < 0.0001) from 1993 to 2012 [2]. The quality of tracheostomy care is likely as important as its timing on the final outcomes of patients [34, 35]. The associated mortality decreased as tracheostomy techniques and postoperative care improved, which caused the inter-group difference in mortality to decrease as well.

In the present study, delayed tracheostomy was associated with longer hospital and ICU stays, duration of MV and sedation, which is consistent with conclusions from previous studies [15–17, 19, 21, 22, 25, 36–38]. Among this mixed ICU population, the majority were diagnosed with neurogenic injury (66.1%) or trauma (16.3%). Comparing with other diagnoses, neurogenic injury was a more common diagnosis for critically ill patients with tracheostomy. Considering that they appear as stable vital signs but difficult decannulation in the process of treatment, attending physicians would take tracheostomy as strategy. Comparing with mixed population, we found similar results in patients with neurogenic injury, trauma, and digestive disorders in a sub-population analysis after stratifying patients according to their main diagnosis. However, for patients in a coma and cardiovascular, respiratory, and neuromuscular diseases, not all inter-group comparisons showed significant differences. The small sample size in those sub-analyses limits the interpretation of the results. In order to gather more evidence, future investigations of tracheostomy should be conducted in large multi-center cohort or certain non-neurogenic group. Prolonged ICU stays and duration of MV put patients at a higher risk of developing VAP, and VAP also hampers patients’ ability to successfully wean off MV and ICU [24, 39]. Chorath et al. and de Franca et al. suggested that ET (≤ 7d) relates with a lower risk of developing VAP, which is inconsistent with the findings described by Terragni et al. [22, 25, 37]. In the present study, VAP was a valuable indicator prompting patients’ poor status, unfortunately, it is difficult to extract the associated information from medical records for now.

Considering the risk of infection that clinicians face during tracheostomy operations, multiple consensus guidelines recommended avoiding or delaying tracheostomy at least until post-intubation day 10 [40, 41]. Ahn et al. proposed that a timely tracheostomy can be conducted regardless of intubation duration or a positive COVID-19 test [42]. In fact, COVID-19 reaches its peak concentration before day 5 and steadily decreased infectivity thereafter [43]. Generally, intubation is applied at 9–10 days after symptom onset [44]. During the time difference between intubation and the onset of symptoms, the infectivity of tracheal secretions decreased. The timing of tracheostomy for patients with COVID-19 attracted a lot of attention, and numerous studies were conducted in this specific population. Some studies revealed that early percutaneous dilatational tracheostomy was safe and could optimize clinical course of patients as well as distribution of critical care resources [45–50]. However, two recent studies investigating patients with COVID-19 reported an increased mortality in patients undergoing ET (< 10d after intubation; < 21d after intubation) [51, 52]. In summary, whether patients with COVID-19 could get clinical benefits by performing early tracheostomy is still controversial, which need more investigations to figure out.

This study has several limitations. Firstly, its retrospective design makes it susceptible to selection and information biases. Secondly, the study included a total of 1884 patients who were admitted to our ICU over a 10-year period. However, due to the inability to contact many of these patients, it was challenging to obtain long-term outcome data, such as follow-up mortality rates, decannulation percentage, and tracheostomy complication rates. Despite our efforts to minimize biases, confounding factors may still exist as a result of the non-randomized nature of this study. Thirdly, the medical policies have changed significantly over a 10-year period, especially during the COVID-19 pandemic, which overwhelmed the healthcare system worldwide. In fact, different countries and regions have implemented varying prevention strategies and control measures. In China, we did not implement open outbreak control measures at the end of 2020, during which COVID-19 patients were admitted to sentinel hospitals. As our hospital was not a sentinel hospital for COVID-19 patients, they were not included in our study, despite our cut-off time for collecting cases being the end of 2020. Currently, there are no specific guidelines or consensus on the timing of tracheostomy for such patients. However, our findings still have value in guiding the timing of tracheotomy in COVID-19 patients who are critically ill.

## Conclusions

In a mixed ICU population, delayed tracheostomy prolonged ICU and hospital stays, sedation durations, and time receiving MV. Similar results were found in sub-population such as neurogenic injury, trauma, and digestive disorders. In order to figure out the correlation between the timing of tracheostomy and outcomes upon discharge, we performed a multinomial logistic regression analysis and delayed tracheostomy (> 10d post-intubation) was independently associated with poor prognosis. Delayed tracheostomy not only cost more medical resources, but also renders prognosis deteriorate. Therefore, our findings were full of clinical value to solve current medical dilemmas.

### Supplementary Information

**Additional file 1: Supplemental Table 1.** Baseline characteristics between ET, IMT and LT. **Supplemental Table 2.** Multinomial logistic regression analysis with outcomes at discharge as dependent variable **Supplemental Figure 1.** Performing situation of tracheostomy of our hospital over past decade. a performing number of tracheostomies per year, (b) in-hospital mortality per year, (c) mean and median time of tracheostomy, (d) distribution of the timing of tracheostomy for included 1884 patients.

## Data Availability

No datasets were generated or analysed during the current study.

## Abbreviations

ARDS: Acute respiratory distress syndrome
APACHE-II: Acute Physiology and Chronic Health Evaluation II
ET: Early tracheostomy
IMT: Intermediate tracheostomy
LT: Late tracheostomy
MV: Mechanical ventilation
VAP: Ventilator-associated pneumonia
ICU: Intensive care unit
RCT: Randomized control study
PDT: Percutaneous dilatational tracheostomy
IQR: Interquartile range
ANOVA: analysis of variance
n: Number
SD: Standard deviation
CI: Confidence Interval
RR: Risk Ratio
COVID-19: Coronavirus disease 2019
