# Supplementary material for: The impact of delayed tracheostomy on critically ill patients receiving mechanical ventilation: a retrospective cohort study in a chinese tertiary hospital
Source: BMC Anesthesiol. 2024 Jan 23;24:39. doi: 10.1186/s12871-024-02411-1 (PMC10804499; doi:10.1186/s12871-024-02411-1)
Supplement: Supplementary file 1 — Additional file 1: Supplemental Table 1. Baseline characteristics between ET, IMT and LT. Supplemental Table 2. Multinomial logistic regression analysis with outcomes at discharge as dependent variable Supplemental Figure 1. Performing situation of tracheostomy of our hospital over past decade. a performing number of tracheostomies per year, (b) in-hospital mortality per year, (c) mean and median time of tracheostomy, (d) distribution of the timing of tracheostomy for included 1884 patients. [file 12871_2024_2411_MOESM1_ESM.docx]

**Supplemental Table 1. Baseline characteristics between ET, IMT and LT**

| Characteristics | Total  n = 1884 | Grouping Criteria I | | | Grouping Criteria II | | |
| --- | --- | --- | --- | --- | --- | --- | --- |
|  |  | ET (≤ 5d)  n = 514 | IMT (5< d ≤10)  n = 729 | LT (> 10d)  n = 641 | ET (≤ 7d)  n = 838 | IMT (7< d ≤14)  n = 710 | LT (>14d)  n = 336 |
| Male, n (%) | 1291(68.5) | 373(72.6) | 487(66.8) | 431(67.2) | 588(70.2) | 464(65.4) | 239(71.1) |
| Age, median, (IQR), year | 65(55-73) | 63(53-76) | 64(55-72) | 67(56-76) | 63(53-71) | 65(56-73) | 70(57-79) |
| Main reasons for admission, n (%) | | | | | | | |
| Neurogenic injury | 1246(66.1) | 359(69.8) | 509(69.8) | 378(59) | 579(69) | 475(66.9) | 192(57.1) |
| Non-traumatic | 1018(54) | 284(55.3) | 420(57.6) | 314(49) | 467(55.7) | 390(54.9) | 161(47.9) |
| Traumatic | 148(7.9) | 51(9.9) | 55(7.5) | 42(6.6) | 72(8.6) | 54(7.6) | 22(6.5) |
| Tumor | 80(4.2) | 24(4.7) | 34(4.7) | 22(3.4) | 40(4.8) | 31(4.4) | 9(2.7) |
| Respiratory disease | 85(4.5) | 13(2.5) | 14(1.9) | 58(9) | 20(2.4) | 26(3.7) | 39(11.6) |
| Cardiovascular disease | 51(2.7) | 16(3.1) | 16(2.2) | 19(3) | 26(3.1) | 13(1.8) | 12(3.6) |
| Coma | 28(1.5) | 11(2.1) | 9(1.2) | 8(1.2) | 15(1.8) | 8(1.1) | 5(1.5) |
| Neuromuscular disease | 33(1.8) | 4(0.8) | 7(1.0) | 22(3.4) | 7(0.8) | 15(2.1) | 11(3.3) |
| Digestive disorder | 53(2.8) | 4(0.8) | 14(1.9) | 35(5.5) | 9(1.1) | 19(2.7) | 25(7.4) |
| Trauma | 308(16.3) | 82(16) | 139(19.1) | 87(13.6) | 148(17.7) | 126(7.7) | 34(10.1) |
| Others | 80(4.2) | 25(4.9) | 21(2.9) | 34(5.3) | 34(4.1) | 28(3.9) | 18(5.4) |
| Type of tracheostomy | | | | | | | |
| Percutaneous | 1168(62) | 377(73.3) | 442(60.6) | 349(54.4) | 596(71.1) | 411(57.9) | 161(47.9) |
| Surgical | 716(38) | 137(26.7) | 287(39.4) | 292(45.6) | 242(28.9) | 299(42.1) | 175(52.1) |

y = year; n = number; IQR = Interquartile Range; SD = Standard Deviation; d = days

**Supplemental Table 2 . Multinomial logistic regression analysis with outcomes at discharge as dependent variable**

| Outcome | Poor Prognosis | | Death | |
| --- | --- | --- | --- | --- |
|  | RR(95%CI)**^a1^** | RR(95%CI)**^a2^** | RR(95%CI)**^b1^** | RR(95%CI)**^b2^** |
| The timing of tracheostomy (from ICU admission, days) | | | | |
| Grouping criteria I | | | | |
| ≤5d | 0.71(0.48,1.06) | NA | 0.88(0.29,2.62) | NA |
| 5<d≤10d | Reference | NA | Reference | NA |
| >10d | 2(1.34,2.97)*** | NA | 1.68(0.7,4.06) | NA |
| Grouping criteria II | | | | |
| ≤7d | NA | 0.66(0.46,0.95)* | NA | 1.61(0.66,3.98) |
| 7<d≤14 | NA | Reference | NA | Reference |
| >14d | NA | 1.95(1.2,3.16)** | NA | 2.22(0.82,6.01) |
| Age (years) | | | | |
| ≤50 | Reference | | | |
| 50-60 | 0.81(0.52,1.27) | 0.8(0.51,1.26) | 2.5(0.5,12.63) | 2.55(0.5,12.89) |
| 60-70 | 0.9(0.59,1.38) | 0.91(0.6,1.4) | 1.36(0.24,7.54) | 1.39(0.25,7.72) |
| 70-80 | 1.16(0.75,1.8) | 1.13(0.73,1.75) | 2.89(0.59,14.2) | 2.91(0.59,14.3) |
| >80 | 0.83(0.48,1.42) | 0.81(0.47,1.4) | 11.4(2.6,50.5)** | 11.8(2.66,52.3) ** |
| Sex | | | | |
| Female | Reference | | | |
| Male | 1.17(0.86,1.58) | 1.14(0.84,1.54) | 1.76(0.81,3.83) | 1.73(0.79,3.79) |
| Duration of stay |  | | | |
| hospital | 1.03(1,1.05)* | 1.03(1,1.05)* | 1.04(1.01,1.08)* | 1.04(1,1.07)* |
| ICU | 0.92(0.89,0.94)*** | 0.92(0.89,0.94)*** | 0.93(0.89,0.97)*** | 0.93(0.9,0.97)*** |
| Type of tracheostomy | | | | |
| Percutaneous | Reference | | | |
| Surgical | 1.39(1.05,1.85)* | 1.34(1.01,1.79)* | 2.1(1.1,4.03)* | 2.16(1.12,4.18) * |
| Length of MV | | | | |
| Total | 1.01(0.98,1.05) | 1.01(0.98,1.05) | 1.06(1.01,1.11)* | 1.06(1.01,1.12)* |
| After TT | 1.06(1.02,1.1)** | 1.06(1.02,1.1)** | 0.99(0.95,1.03) | 0.98(0.94,1.03) |
| Length of sedation | 1.01(1,1.03) | 1.01(1,1.03)* | 1.02(1,1.04)* | 1.02(1.01,1.04)* |

Multinomial logistic regression analysis with outcomes (good prognosis, poor prognosis and death) at discharge as dependent variable. The timing of tracheostomy was measured by duration after ICU admission. The outcomes includes good prognosis, poor prognosis and death, in which good prognosis was the reference. RR**^a1^** indicate the risk ratio of poor prognosis, whereas RR**^b1^** suggest the risk ratio of death (the timing of tracheostomy was grouped by grouping criteria I). RR**^a2^** indicate the risk ratio of poor prognosis, whereas RR**^b2^** suggest the risk ratio of death (the timing of tracheostomy was grouped by grouping criteria II). y = year; d = day; w = week; ICU = Intensive care unit; MV = mechanical ventilation; RR=risk ratio; CI = Confidence Interval.

**Supplemental Figure legend**

Supplemental Figure 1: Performing situation of tracheostomy of our hospital over past decade. (a) performing number of tracheostomies per year, (b) in-hospital mortality per year, (c) mean and median time of tracheostomy, (d) distribution of the timing of tracheostomy for included 1884 patients
